# Supplementary material for: Avoided metallicity in a hole-doped Mott insulator on a triangular lattice
Source: Nat Commun. 2024 Sep 16;15:8098. doi: 10.1038/s41467-024-52007-z (PMC11405841; doi:10.1038/s41467-024-52007-z)
Supplement: Supplementary file 1 — Supplementary Information [file 41467_2024_52007_MOESM1_ESM.pdf]

Supporting Information for ”Avoided metallicity in a hole-doped Mott insulator on a triangular lattice”

Chi Ming Yim*,[1,2,†] Gesa-R. Siemann*,[1] Srdjan Stavrić*,[3,4] Seunghyun Khim,[5] Izidor Benedičič,[1] Philip A. E. Murgatroyd,[1] Tommaso Antonelli,[1] Matthew D. Watson,[6] Andrew P. Mackenzie,[1,5] Silvia Picozzi,[3,‡] Phil D.C. King,[1,§] and Peter Wahl[1,7,¶]

[1]*SUPA, School of Physics and Astronomy, University of St Andrews, North Haugh, St Andrews, Fife, KY16 9SS, United Kingdom*

[2]*Tsung Dao Lee Institute and School of Physics and Astronomy, Shanghai Jiao Tong University, Shanghai, 201210, China*

[3]*Consiglio Nazionale delle Ricerche (CNR-SPIN), Unità di Ricerca presso Terzi c/o Università “G. D’Annunzio”, 66100 Chieti, Italy*

[4]*Vinča Institute of Nuclear Sciences - National Institute of the Republic of Serbia, University of Belgrade, P. O. Box 522, RS-11001 Belgrade, Serbia*

[5]*Max Planck Institute for Chemical Physics of Solids, Nöthnitzer Straße 40, 01187 Dresden, Germany*

[6]*Diamond Light Source, Harwell Science and Innovation Campus, Didcot, OX11 ODE, United Kingdom*

[7]*Physikalisches Institut, Universität Bonn, Nussallee 12, 53115 Bonn, Germany*

(Dated: August 12, 2024)

* These authors contributed equally.

[†]Electronic address: c.m.yim@sjtu.edu.cn.
[‡]Electronic address: silvia.picozzi@spin.cnr.it
[§]Electronic address: pdk6@st-andrews.ac.uk
[¶]Electronic address: wahl@st-andrews.ac.uk

Supplementary note 1. Determination of the surface work function of the CrO_2-terminated surface

We have measured the image potential states of the CrO_2-terminated surface, achieved by measuring the differential conductance $g(V)$ as a function of bias voltage V in a closed loop condition. Fig. S1 shows such a $g(V)$ spectrum recorded from a defect-free position of the CrO_2-terminated surface, in which the position of the lowest order peak ($n = 0$) can be taken as an approximation of the surface work function. We have therefore determined that the CrO_2-terminated surface has a surface work function of $7.4\mathrm{eV}$, very close to that of the CoO_2 termination of $PdCoO_2$.[1]

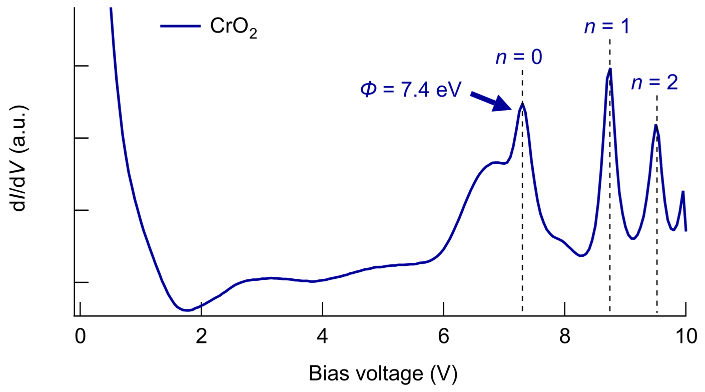

Supplementary Figure S1: **Measurement of work function of the CrO_2- terminated surface of $PdCrO_2$.** Point $g(V)$ spectrum recorded with closed feedback-loop from the CrO_2 surface termination ($I_s = 10\mathrm{pA}$; $V_m = 50\mathrm{mV}$). The arrow marks the lowest order peak of the standing-wave states, the energy position of which approximates the surface work function ϕ.

Supplementary note 2. Surface homogeneity

While cleaving the samples results in areas with different surface terminations and could, in principle, also result in inhomogeneity with each surface termination, from our data as well as experience with measurements on the surfaces of other delafossite oxides we have ample evidence that the areas we have investigated are dominated by a single homogeneous termination. We have extensively characterised the distinct terminations of the cleaved surfaces of the family of $(\mathrm{Pd}, \mathrm{Pt})(\mathrm{Co}, \mathrm{Cr})O_2$ compounds. In the Co-based systems, extremely clear signatures of

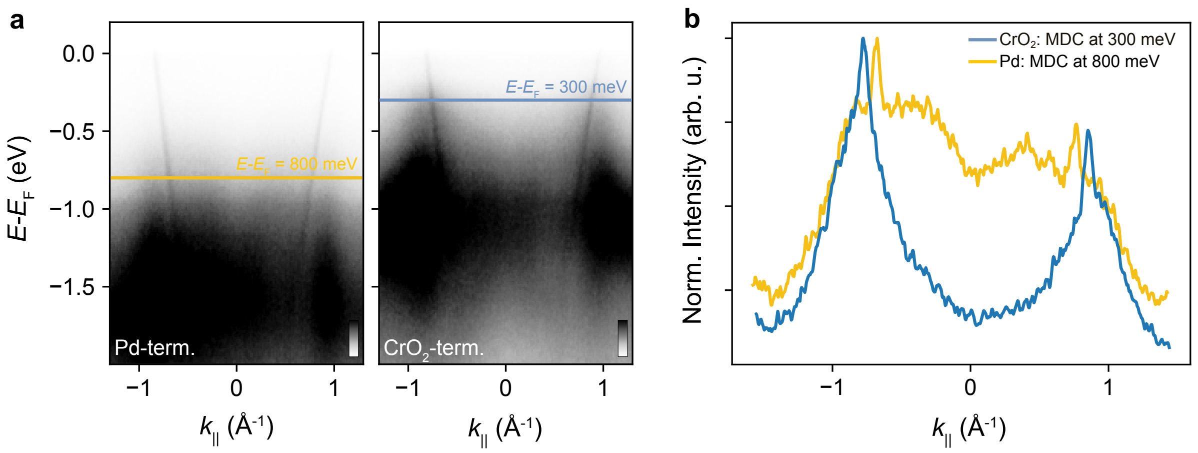

Supplementary Figure S2: **Termination-dependent ARPES measurements.** Comparison of the extracted momentum distribution curves (MDCs) from the two surface regions (labelled Pd- and CrO_2 in (a) at different binding energies ($E - E_F$) as indicated by the solid lines in the spectra shown in (a). Clear differences can be seen the in the extracted MDCs separated by an energy difference of 500 meV, ruling out that these spectra from the different surface terminations can be understood simply via a rigid band shift.

electron- and hole- doping of the CoO_2 and (Pd, Pt) surface terminations can be observed, with clear-cut spectral signatures that easily allow identifying any minority/mixed terminations[2,3], and again consistent between ARPES and STM[1,4]. In such measurements, we have observed well-defined surface terminations with length scales $\gg 50\mu m$, entirely consistent with the length scale of the variations that we assign here for the CrO_2- and Pd-terminated surfaces. With similar variations also in core-level spectra, and spectra that cannot be simply reproduced via a rigid band shift (Supplementary Figure S2), we are confident in similarly assigning the observed patches here as Pd- and CrO_2- terminated regions, with minimal contribution from charge puddles due to excess Pd impurities. This does not mean that the surface terminations are atomically flat over this probing region. Indeed, large scale topographic images as shown in Figure S3(a) are characterised by flat terraces separated by step edges running along the same direction. The line profile taken across the terraces shows clearly that the terraces are separated from each other by the same step height of $\sim 600 pm$, indicating that all the terraces are of the same (here CrO_2) termination. Importantly, there is negligible sign within the probing region of spatially varying inhomogeneity as can be seen in the negative Laplacian of the topography

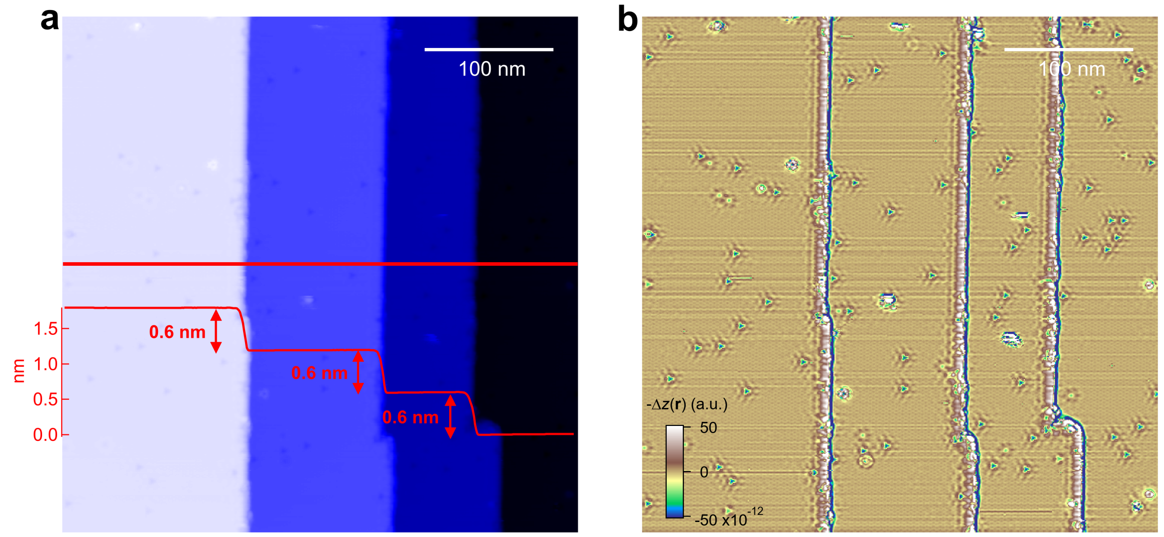

Supplementary Figure S3: **Large-scale STM image.** (a) STM image with a lateral size of $400 \times 400 \mathrm{nm}^2$, showing multiple terraces with the same CrO_2 termination and a very high surface homogeneity ($V = 1.5\mathrm{V}$, $I = 0.1\mathrm{nA}$). (b) Negative Laplacian of the topographic image in (a).

in Figure S3(b).

Supplementary note 3. Sub-surface charge transfer

The small changes in k_{F} evident in fig. 2c of the main text for the bulk-like Pd states measured at the Pd vs. the CrO_2-terminated surface point to a small charge transfer from the CrO_2-termianting surface to the subsurface Pd layer. In Figure S4a, we reproduce the spatial variations in k_{F} of the Pd-derived bulk bands, illustrating a reduced k_{F} on a CrO_2 terminated surface as compared to the Pd-terminated areas. To allow for a direct comparison between the Pd-derived states probed on the two surface terminations, we plot the resulting peak positions from fits to the MDCs of the Pd-derived states shifted by their respective k_{F} (blue and yellow lines) in Figure S4b. The resulting Fermi velocity (v_{F}) of these states, fitted using a linear approximation, is plotted on top of the data (red and purple solid lines). For the CrO_2-terminated surface we find a $v_{\mathrm{F}} = 4.2 \pm 0.1 \mathrm{eV\AA}$ which is slightly smaller than for the Pd-terminated surface where $v_{\mathrm{F}} = 4.6 \pm 0.1 \mathrm{eV\AA}$. The lowering of v_{F} for the CrO_2-terminated surface is in agreement with our calculations which show a small charge transfer between the surface CrO_2

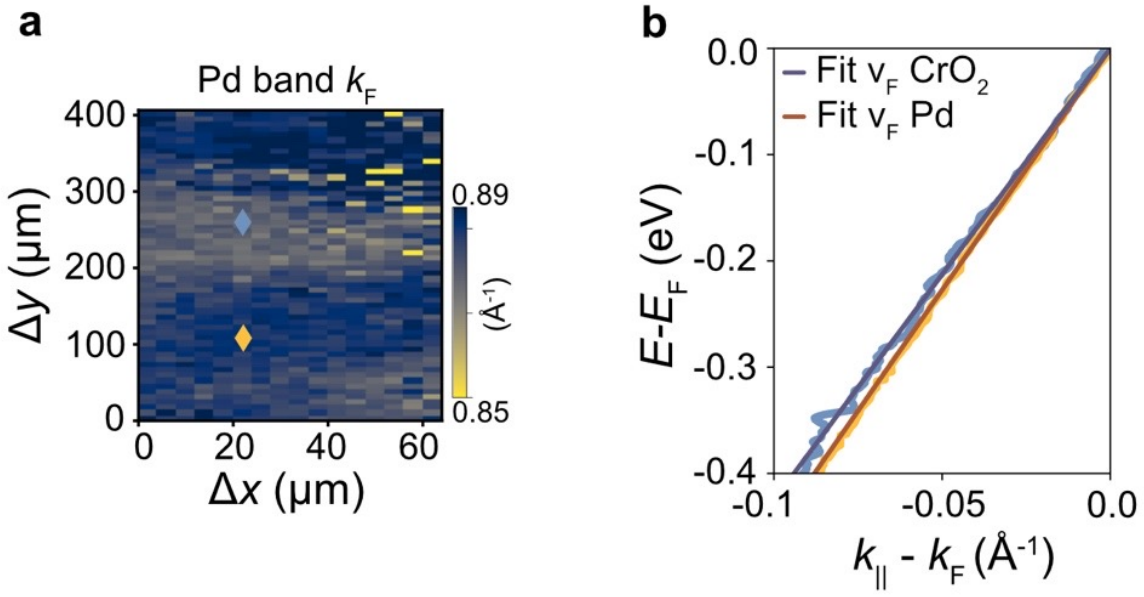

Supplementary Figure S4: **Termination-dependent variations of k_F.** (a) spatial map of the Fermi wave vector as shown in fig. 2c of the main text. (b) linear fits to the dispersion relation from which the Fermi wave vector has been extracted. Small but systematic deviations can be seen in the Fermi velocity.

and the subsurface Pd layer (Fig S13). Such a charge transfer indicates an enhanced coupling between the Pd and Cr-derived states. This can be expected to lead to a reduced k_F (slight hole-doping on the subsurface Pd layer) and reduced v_F (due to the increase in Cr character in these states) of the subsurface Pd states measured here. This is entirely consistent with our experimental findings shown in Figure S4.

Supplementary note 4. Comparison of tunneling spectra with photoemission

In fig. S5, we show additional data highlighting the differences in photoemission between Pd- and CrO_2-terminated patches, as well as a detailed comparison between photoemission and tunneling spectroscopy. From the photoemission data (fig. S5a), we observe in the data of the CrO_2 terminated surface clear development of a low-energy shoulder at a binding energy of approximately $-0.5\mathrm{eV}$ (S-Cr), which is completely absent in the data of the Pd terminated surface. This S-Cr shoulder is also evident in the momentum-integrated data in fig. S5b, where it shows up as a change in the slope of the curve in the vicinity of the Fermi level, and can be clearly resolved in peak fits to the data. Specifically, through peak fitting to the EDC using

one component for the S-Cr shoulder and other components for peaks at higher binding energy we determined the binding energy of the S-Cr peak to be approximately $-0.44\mathrm{eV}$. This value is in good agreement with the peak observed in our differential conductance spectra recorded with STM. In fig. S5(c), we show a fit to the peak at the gap edge in the tunneling spectrum, and determine an energy of $-0.43\mathrm{eV}$, very close to the energy of the S-Cr component found in photoemission. We conclude that in both STM and ARPES we have observed independent evidence for the surface gap of the CrO_2 termination.

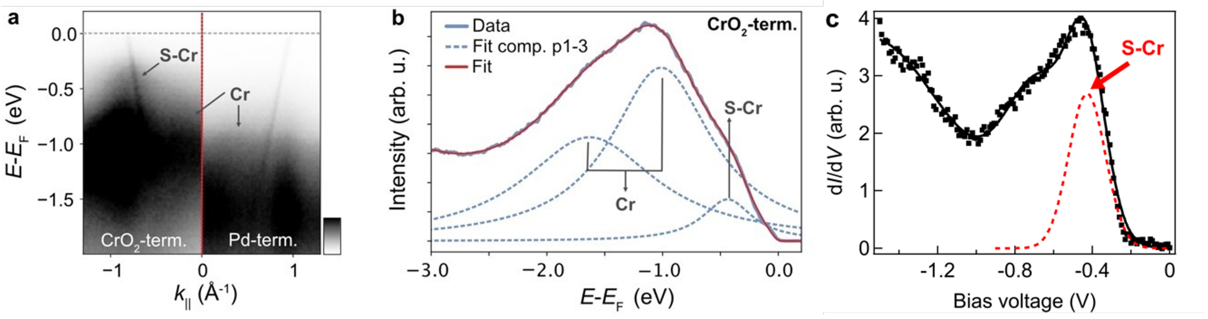

Supplementary Figure S5: **Comparison of tunneling spectra and photoemission.** **a** High resolution ARPES data of a CrO_2- (left) and Pd-terminated (right) area of the sample. Both spectra have been probed using a photon energy of $110\mathrm{eV}$ and LH polarised light. **b** The k-integrated spectra of the CrO_2-terminated data extracted from a. By fitting the data, a clear multi-peak structure is revealed (see fit components 1-3, blue dashed lines) which is attributed to the presence of the bulk components of the Cr-derived states (labelled Cr) as well as the formation of a surface component (S-Cr), which results in the observed shoulder in the k-integrated data. **c** STS data (black dots) of the CrO_2 terminated surface of the sample taken within the sample bias range between $-1.5\mathrm{V}$ and $+1.5\mathrm{V}$. ($V_s = 1.5\mathrm{V}$, $I_s = 50\mathrm{pA}$, $V_{\mathrm{mod}} = 10\mathrm{mV}$). Only data points for negative bias voltages, i.e. in the occupied states, are shown. Black line: numerical fit to the STS data. Red dotted line: peak associated with the Cr-derived surface states ($E = -0.43\mathrm{eV}$).

Supplementary note 5. Bias-dependence of STM imaging and spectroscopy of charge order

We have imaged the CrO_2-terminated surface of $PdCrO_2$ at energies both outside ($V = 350\mathrm{mV}$) and inside the gap ($V = 150\mathrm{mV}$). Shown in Fig. S6, the point defects in the CrO_2 surface layer are clearly visible at $V = 350\mathrm{mV}$; however, they become virtually invisible when

imaged at energies inside the energy gap.

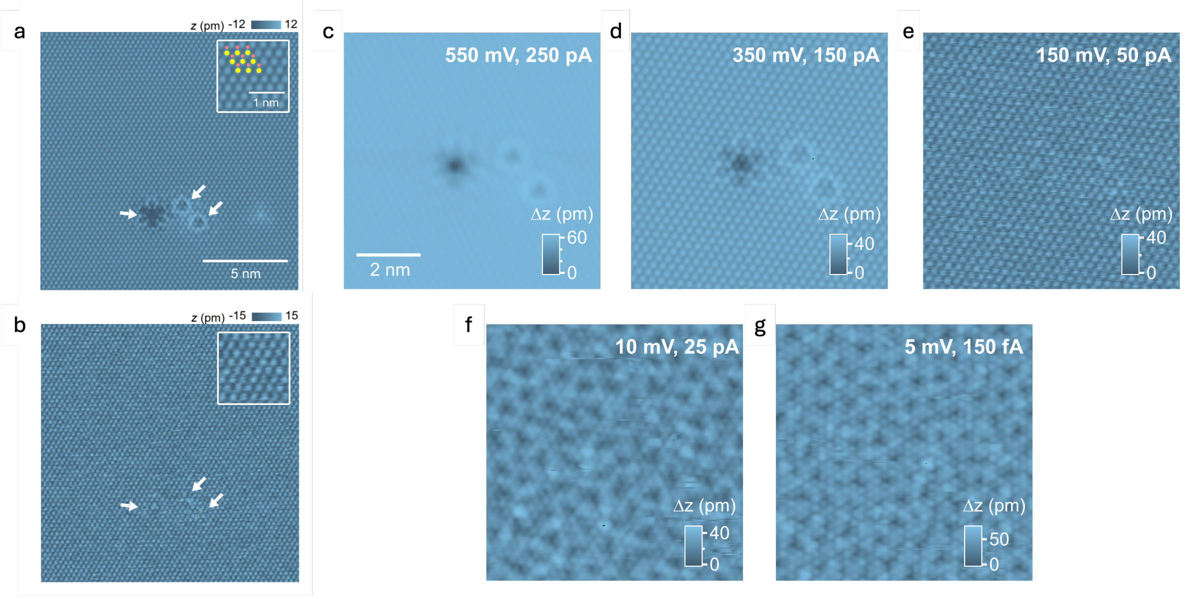

Supplementary Figure S6: **Bias voltage dependence of the appearance of point defects in the CrO_2 terminated surface.** Atomically resolved STM topographs of the CrO_2-terminated surface taken at scan parameters of (**a**) $V = 350\text{mV}, I = 150\text{pA}$ and (**b**) $V = 150\text{mV}, I = 50\text{pA}$ respectively. Image size: $(30\text{nm})^2$. The images show the point defects (indicated by white arrows) become virtually invisible when imaged at bias voltages V inside the gap. **c**-**e**, Images taken at the same surface location at different sample biases and tunneling currents (V, I): (c) 550mV, 250pA, (d) 350mV, 105pA, (e) 150mV, 50pA. Image size: $(8\text{nm})^2$. As in a and b, the surface point defects showing up in the high sample bias images (c and d) become barely visible in the image taken at 150mV (e). Images taken in this bias range ($150 - 550\text{mV}$) show perfect triangular lattice of the CrO_2 surface. **f**, **g**, Images taken at energies in the vicinity of the Fermi level. Image size: $(8\text{nm})^2$. The image in (f) was recorded at $V = 10\text{mV}$, $I = 25\text{pA}$, while that in (g) was taken with much lower tunneling current ($V = 5\text{mV}$, $I = 150\text{fA}$). Note that the images in (f) and (g) were recorded using different frame angles. Both images show the glassy structure. However, due to the much lower current used, the image in (g) shows much fewer streaks.

The unusual changes in the appearance of topographic images with bias voltage V and current I are further highlighted in fig. S6c-g. The images taken in the sample bias range between 150mV and 550mV (fig. S6c-e) show a perfect triangular lattice with no evidence of the order however some evidence of fluctuations, e.g. in panel e where some streaky appearance can be seen. The image contrast becomes drastically different when the sample bias is decreased to

values of only a few millivolts and small currents. As shown in fig. S6f, g, the images taken at $10\mathrm{mV}$ or below exhibit the short-range ordered glassy structure instead of the perfect triangular structure. In addition, at roughly fixed sample bias $(5-10\mathrm{mV})$, when lowering the current by ~ 150 times $(25\mathrm{pA} \rightarrow 0.15\mathrm{pA})$, the streaks occasionally present in (f) become much less frequent in appearance in (g). All these images indicate that the perfect triangular lattice of the CrO_2 surface shows up when the surface is imaged at energies outside the surface gap and the glassy structure dominates when imaged at energies inside the gap. In addition, in order to image the glassy structure without any tip-induced disruption to the structure, imaging of the surface needs to be performed at very low sample bias (a few mVs) and extremely low tunnelling currents $(\sim 200\mathrm{fA})$. This at the same time indicates that the characteristic energy scale associated with any excitations taking place in the glassy structure is in the few milli-electronvolt range.

In Fig. S7, we show tunneling spectra obtained on bright and dark atoms on the CrO_2 surface. There are clear differences in the shoulders around $+/-5\mathrm{meV}$.

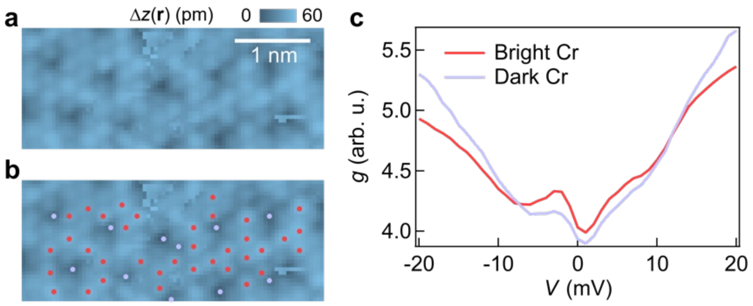

Supplementary Figure S7: **Tunnelling spectroscopy on the CrO_2 terminated surface.** a, atomically resolved STM topographic image taken from the CrO_2-terminated surface of $PdCrO_2$ ($V = 20\mathrm{mV}$, $I = 30\mathrm{pA}$, Size = $4 \times 1.5\mathrm{nm}^2$). b, As (a), with overlaid dots of different colours marking the positions of the ' bright ' (red) and ' dark ' (light blue) Cr ions at which point differential conductance spectra $g(V)$ were taken. c, Averaged $g(V)$ spectra taken from the 'bright' (red) and 'dark' (light blue) Cr atoms. Spectroscopy setpoint $V_s = 20\mathrm{mV}$, $I_s = 100\mathrm{pA}$. Frequency and amplitude of bias modulation used $f_m = 413\mathrm{Hz}$, $V_m = 1\mathrm{mV}$.

Supplementary note 6. Calibration of the DFT+U computational approach

Due to the correlated nature of the electronic states in the delafossite $PdCrO_2$, their realistic modelling requires corrections that account for these correlations beyond conventional DFT. Here, we include these by undertaking calculations in the DFT+U approach. We have performed extensive tests of the DFT+U approach and compared the results both to experimental data and to calculations using DFT+DMFT to verify the fidelity in describing the correlated band structure. We have modelled bulk $PdCrO_2$ by using a $\sqrt{3} \times \sqrt{3}$ supercell with 6 layers, as described in the Methods section of the main text. We have performed calculations accounting for the noncollinear spin configuration of the antiferromagnetic (AF) noncoplanar $120°$ spin configuration (model 4 in ref.[5]) depicted in Fig. S8c. For different Hubbard values $U = 2, 3, 4\,\mathrm{eV}$ and fixed Hund's coupling $J = 0.9\,\mathrm{eV}$, we have performed self-consistent field calculations and projected the density of states onto the Cr d orbitals (PDOS). The PDOS obtained from DFT+U calculations is compared to the PDOS extracted from resonant photoemission measurements (Fig. 2c of Ref.[6]). As evident from Fig. S8a, $U = 4\,\mathrm{eV}$ and $J = 0.9\,\mathrm{eV}$ yield the best agreement between the DFT-calculated and measured PDOS. Therefore, we used these values in all our DFT+U calculations.

To inspect how the particular AF spin configuration influences the electronic structure, we calculated the band structure of the "model 4" and "model 3" spin configurations from Ref.[5]. The total energies of the two configurations obtained from DFT+U are very similar, with "model 4" being only $0.7\,\mathrm{meV}$ per Cr atom lower in energy compared to "model 3". These spin configurations differ in relative orientation of the $120°$ spin planes in subsequent CrO_2 layers. The band structures corresponding to two spin configurations are unfolded to the Brillouin zone of the primitive cell (Fig. S8b) and depicted in Fig. S8c and d, with the corresponding spin configurations displayed on the left of each panel. As these band plots are very similar in a wide energy range, we conclude that the magnetic coupling between adjacent CrO_2 layers has only a minor effect on the overall electronic structure of the system.

Compared to the electronic structure calculated with DMFT[6–8], the DFT+U approach with $U = 4\,\mathrm{eV}$ is performing quite well. By comparing the Pd-derived bands from Fig. S8c with the DMFT calculations shown in Fig. S8e and f, the single dispersive band crossing E_F is very

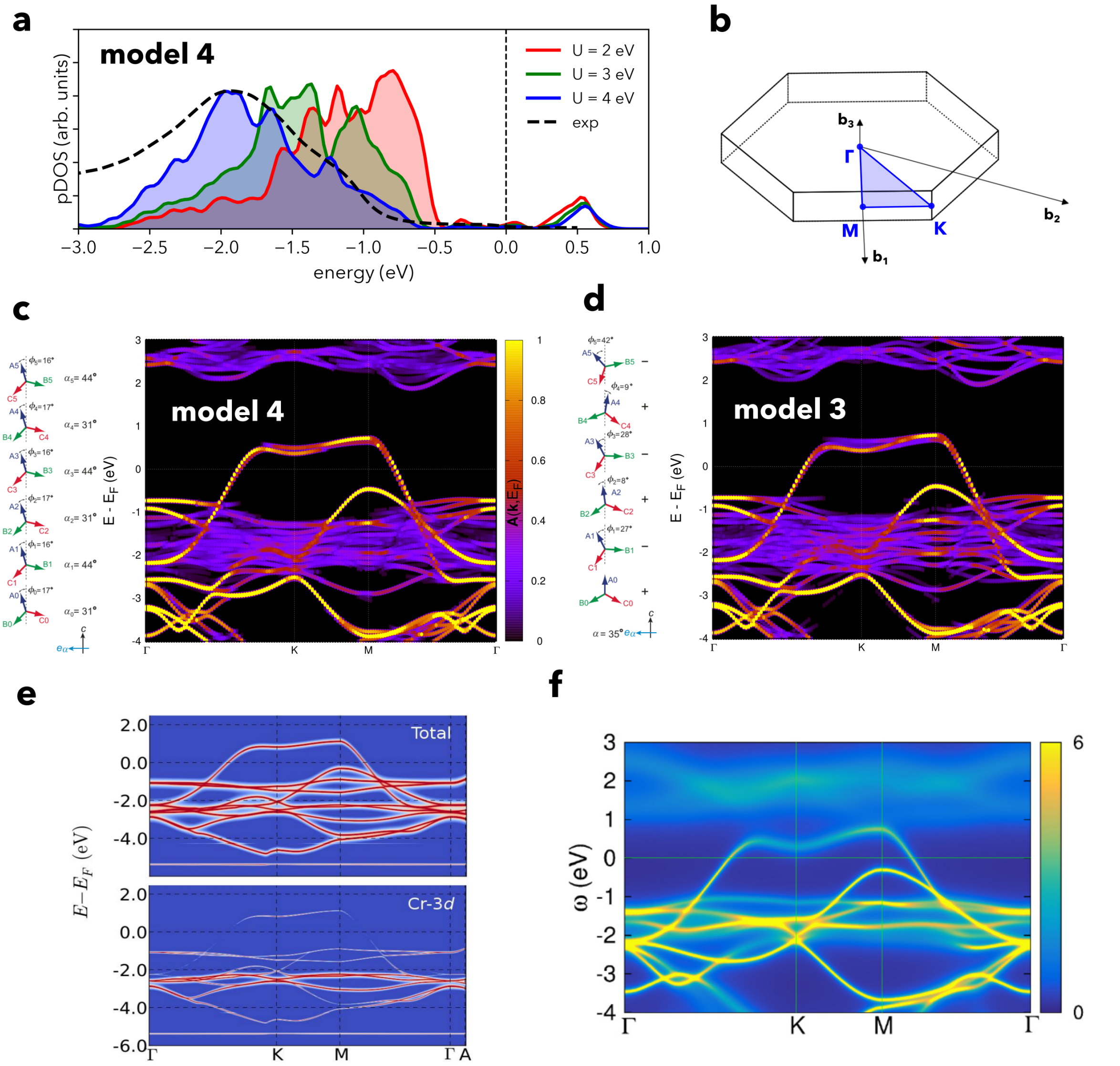

Supplementary Figure S8: **Bulk electronic structure of $PdCrO_2$.** (**a**) Projected density of states (PDOS) of Cr $3d$ orbitals obtained from DFT+U calculations, including relativistic corrections and noncollinear spin order, for different U and $J = 0.9\,\text{eV}$, compared to the PDOS extracted from the photoemission measurements in Ref.[6] (black dashed line). (**b**) Brillouin zone of the primitive cell. (**c**, **d**) Effective band structures, unfolded to the Brillouin zone of the primitive cell, for the "model 4" and "model 3" spin configurations taken from Ref.[5]. Panels to the left depict the spin configurations (adapted from Fig. 5 of Ref.[5]). The band structure from DMFT calculations is shown in panels **e**[6] and **f**[7] for comparison. Models in panel c, d reprinted with permission from Ref.[5], Copyright (2014) by the American Physical Society.

similar in the two approaches, and so is the band reaching its maximum at the M point just be-

low E_F. The band splitting observable in the DFT+U band structure at Γ slightly above -1 eV (Fig. S8c) is due to spin-orbit coupling (SOC). We expect that this splitting is not observable in Fig. S8e and f since SOC was not included in the DMFT calculations. Concerning the Cr-derived bands, DMFT shows them lying in the range from -2.5 eV to -1 eV. This agrees with our DFT+U band structure for $U = 4$ eV. The choice of U is crucial for the position of these bands, with higher U values shifting them towards higher binding energies.

With U fixed at 4 eV, we performed the calculations on bulk $PdCrO_2$ in three different ways: (1) by using the $\sqrt{3} \times \sqrt{3}$ unit cell and the "model 4" AF-120 spin configuration within the non-collinear DFT+U approach and including spin-orbit coupling, (2) using the same AF-120 spin configuration within non-collinear DFT+U but without spin-orbit coupling and (3) by using the $\sqrt{7} \times \sqrt{7}$ unit cell and the "4-up 3-down" collinear spin configuration within the spin polarised DFT+U approach (see Methods section of the main text). The PDOS plots obtained from the two approaches are displayed in Fig. S9. Firstly, from Fig. S9a, we conclude that

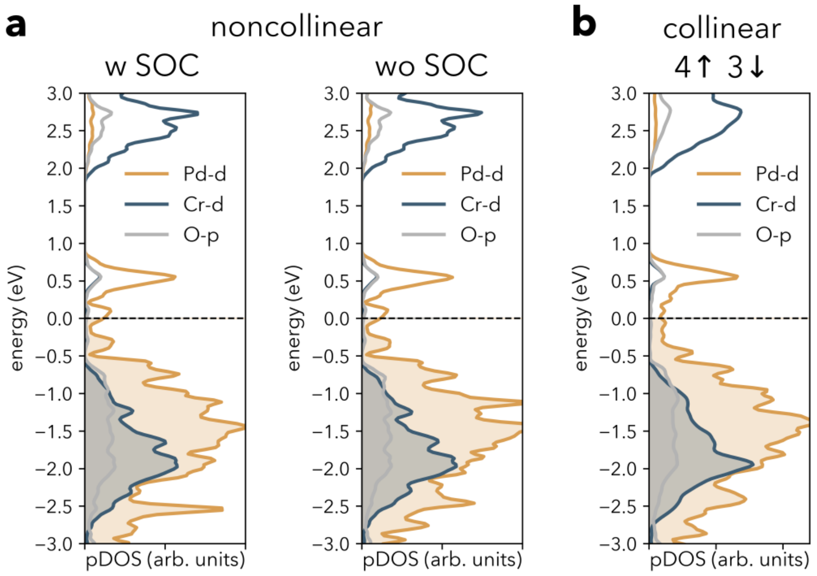

Supplementary Figure S9: **PDOS of bulk $PdCrO_2$ from three different approaches.** (**a**) PDOS of noncollinear "model 4" AF-120 spin configuration calculated using the $\sqrt{3} \times \sqrt{3}$ unit cell with spin-orbit coupling (SOC) (left) and without SOC (right). (**b**) PDOS calculated within spin-polarised DFT+U approach using the $\sqrt{7} \times \sqrt{7}$ unit cell with four layers, with 4 and 3 opposite spins in collinear configuration distributed among the layers in an alternating way as described in the Methods section of the main text.

SOC plays a minor role in the electronic structure of $PdCrO_2$, especially for the Cr-derived states. Secondly, the PDOS obtained from collinear calculations, Fig. S9b, looks very similar to that from noncollinear calculations, with very similar band widths in the two cases and major resonances showing up at the same energies. Therefore, we conclude that for treating of the CrO_2 surface we can neglect SOC and turn to the computationally much more affordable spin-polarised DFT calculations.

Supplementary note 7. Simulated STM images of the CrO_2 surface

For comparison, we provide in Fig. S10 simulated STM images which show that indeed the reconstructed CrO_2 surface reveals patterns that look similar to the experimentally observed ones obtained at very low bias voltages. We note that in experiment, the surface structure cannot be imaged with bias voltages $|V| > 20\mathrm{mV}$, because the tunneling electrons induce changes in the order in the surface layer.

By changing the bias voltage in DFT simulations, we are able to capture the most prominent features of experimental STM images: at larger bias voltages ($V = 200\mathrm{mV}$, Fig. S10a) a perfect triangular lattice can be observed, with no evidence of the $\sqrt{7} \times \sqrt{7}$ reconstruction. On the other hand, at small bias voltages, when only the Pd-derived electronic states close to the Fermi energy and located inside the surface gap are probed (Fig. S10b,c), we clearly observe a difference in contrast due to the two types of Cr atoms, with a spatial variation that is consistent with the local contrast observed in our measurements. We note that the contrast variation is more inhomogeneous in our experiments, however, consistent with the glassy nature of this state. Note that the STM images do not capture the Cr states directly (these states are gapped away) but instead the subsurface Pd states by tunnelling through the CrO_2 layer.

Supplementary note 8. Influence of the atomic relaxation and spin configuration on the band gap in surface CrO_2 layer

In the main text we concluded that the surface relaxation is necessary for the band gap opening in the CrO_2 layer. To demonstrate this we performed additional calculations for the unrelaxed surface, assuming bulk-like atomic positions of Cr atoms and the FM spin configura-

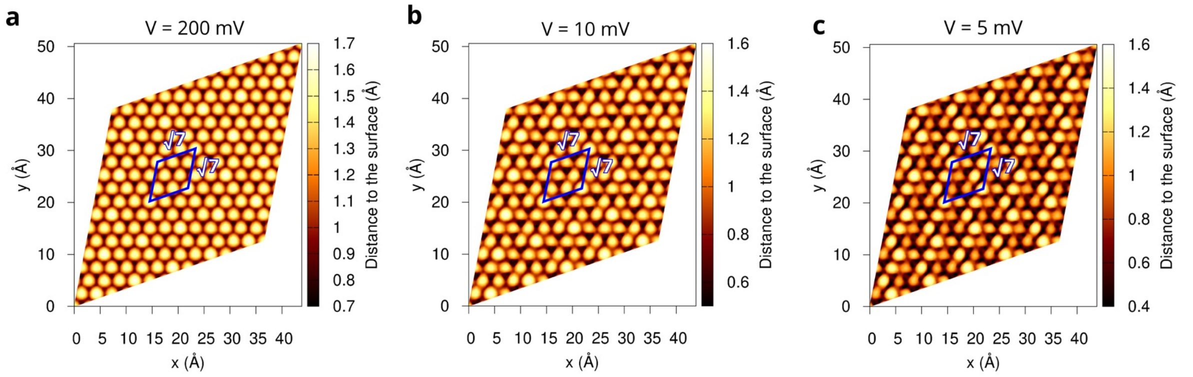

Supplementary Figure S10: **Simulated STM images.** Simulated STM images obtained in constant-current mode at (a) $V = 200\,\mathrm{mV}$, showing a perfectly triangular lattice, and (b, c) at $10\,\mathrm{mV}$ and $5\,\mathrm{mV}$, respectively, showing the $\sqrt{7} \times \sqrt{7}$ reconstruction.

tion. The PDOS plots in Fig. S11b clearly show the metallic surface, with Cr $3d$ states giving a dominant contribution in the whole energy interval from -0.4 to $0.4\,\mathrm{eV}$ around E_F. As the Cr atoms from the unrelaxed surface are symmetry-equivalent, there is no mechanism that would allow their division into two distinct types and the charge disproportionation does not occur.

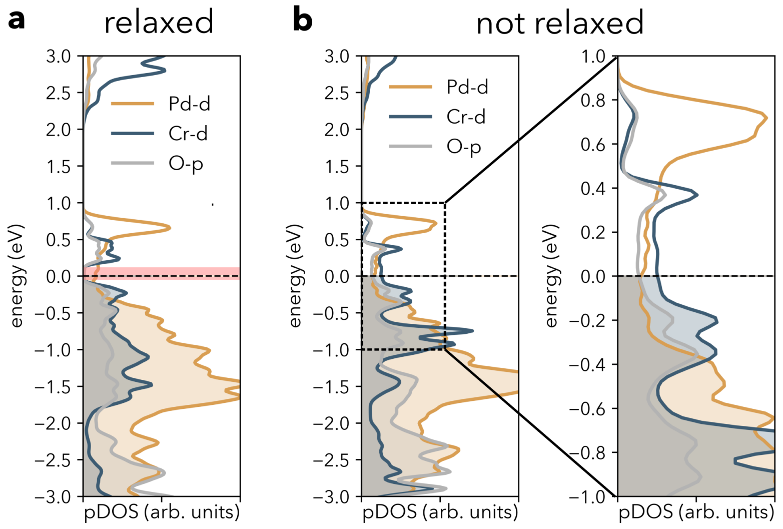

Supplementary Figure S11: **Effect of surface relaxation on the gap opening**. (a) PDOS for relaxed surface. (b) PDOS for unrelaxed surface with ferromagnetic order, including a zoom-in onto the energy range around the Fermi energy. No band gap is observed. The band gap opens only after the surface is relaxed. Surface relaxation, starting from positions inherited from the bulk, lowers the total energy by 160 meV/Cr.

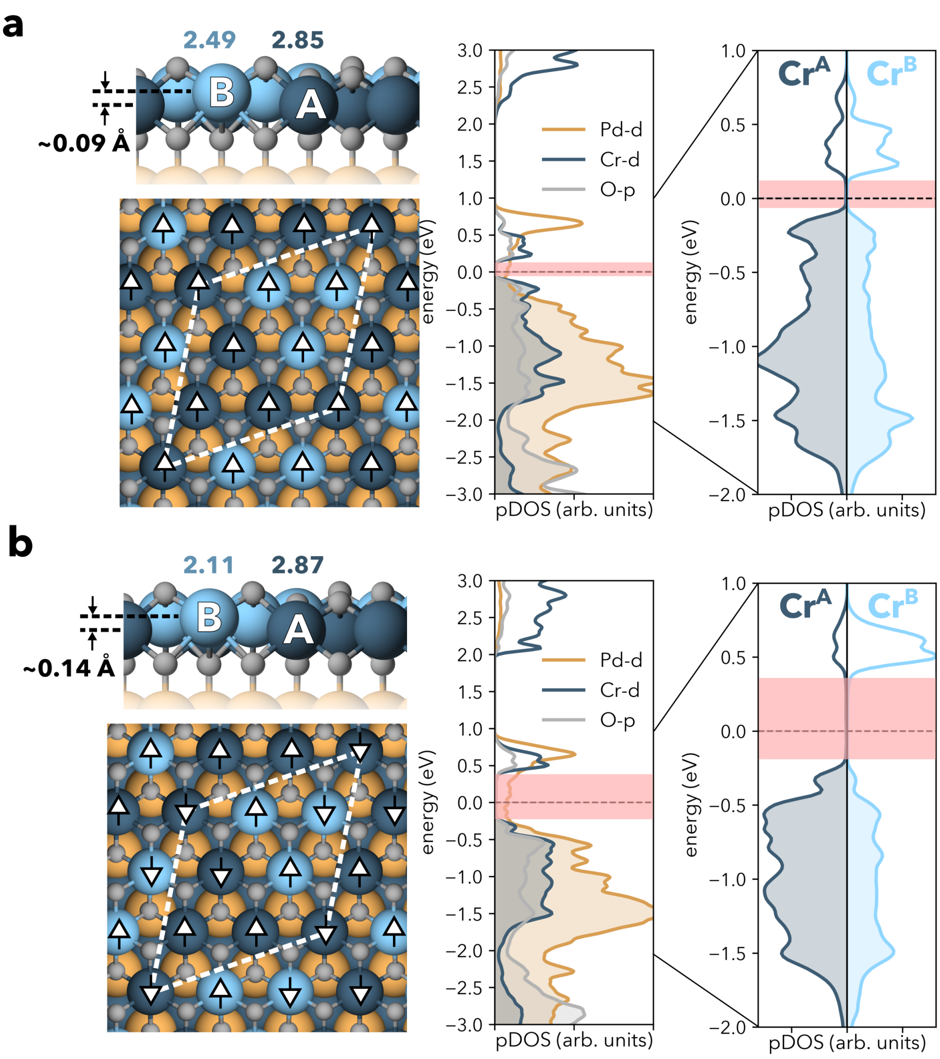

Supplementary Figure S12: **Spin configuration at the surface and its relation to the size of the gap.** (**a**) Ferromagnetic configuration from the main text and (**b**) the ”4-up 3-down” configuration. The type of Cr atoms is not related to the magnetic moment direction, i.e. there are both spin-up and spin-down Cr^A (Cr^B) atoms.

The surface spin configuration, although not responsible for the band gap opening, can affect its size. To shed light on its influence, we performed surface relaxations with a $\sqrt{7} \times \sqrt{7}$ unit cell starting from a spin configuration with 4 spin-up and 3 spin-down Cr atoms that we refer to as ”4-up 3-down” spin configuration (Fig. S12). After the relaxation, the structure ended in a local minimum that is 2.6 meV/Cr higher in energy than the ferromagnetic surface but which has some features, apart from the magnetic order, clearly distinguishing this metastable state from the ferromagnetic ground state. Firstly, the band gap is much larger ($\sim 0.6\,\mathrm{eV}$) compared

to the ferromagnetic surface ($\sim 0.2\,\text{eV}$). This is a joint effect of the absence of FM exchange in the ”4-up 3-down” state and of the more pronounced surface reconstruction, as evidenced by larger displacement of Cr^{B} atoms as compared to the FM state (Fig. S12b, left panel). It is worth noting that in the ”4-up 3-down” configuration the projection of the magnetic moment is *not* related to the type of Cr atom, i.e. there are both spin-up and spin-down magnetic moments among both Cr types. However, the magnitude of the magnetic moment is tied to the Cr type, with Cr^{A} (Cr^{B}) always having the magnetic moments with magnitude of 2.87 (2.11) μ_B.

Supplementary note 9. Layer-resolved electronic band structure

To analyze charge transfer to the Pd-layers, we have determined the band structure from spin-polarised DFT+U calculations in the $\sqrt{7} \times \sqrt{7}$ unit cell, unfolded them to the primitive cell (Brillouin zone shown in Fig. S8b) and projected onto the different layers. The Pd-derived band crossing the Fermi energy E_{F} in the bulk is in the topmost Pd layer moved by $\sim 0.1\,\text{eV}$ towards lower binding energies as compared to the bulk (Fig. S13a, b). The same shift can be observed for the band maximum at the M point. This band shift suggests that the subsurface Pd layer is hole doped compared to the bulk.

For completeness, we show the CrO_2-bands, with contributions from Cr and O atoms summed up. We can clearly see that in the energy range from $-1\,\text{eV}$ to $1\,\text{eV}$ there is only the contribution from the surface layer, as the bulk CrO_2 bands are at higher binding energies (Fig. S13c, d). We further projected the band structure of the CrO_2 surface on two types of Cr atoms. Evidently, Cr^{A} (Cr^{B}) atoms contribute more than Cr^{B} (Cr^{A}) atoms in the energy window below (above) the E_{F}, which is consistent with the PDOS plot of Fig. 3c of the main text showing that Cr^{B} atoms are more strongly hole doped.

Supplementary note 10. Imaging of the charge-disproportionated order

To demonstrate the sensitivity of the charge-ordered insulating state to the tunneling conditions chosen, we first imaged the order at $V = 5\text{mV}$, then at a higher bias voltage of 200mV, and then at $V = 5\text{mV}$ again. The obtained images are shown in Fig. S14, a to c. To demonstrate

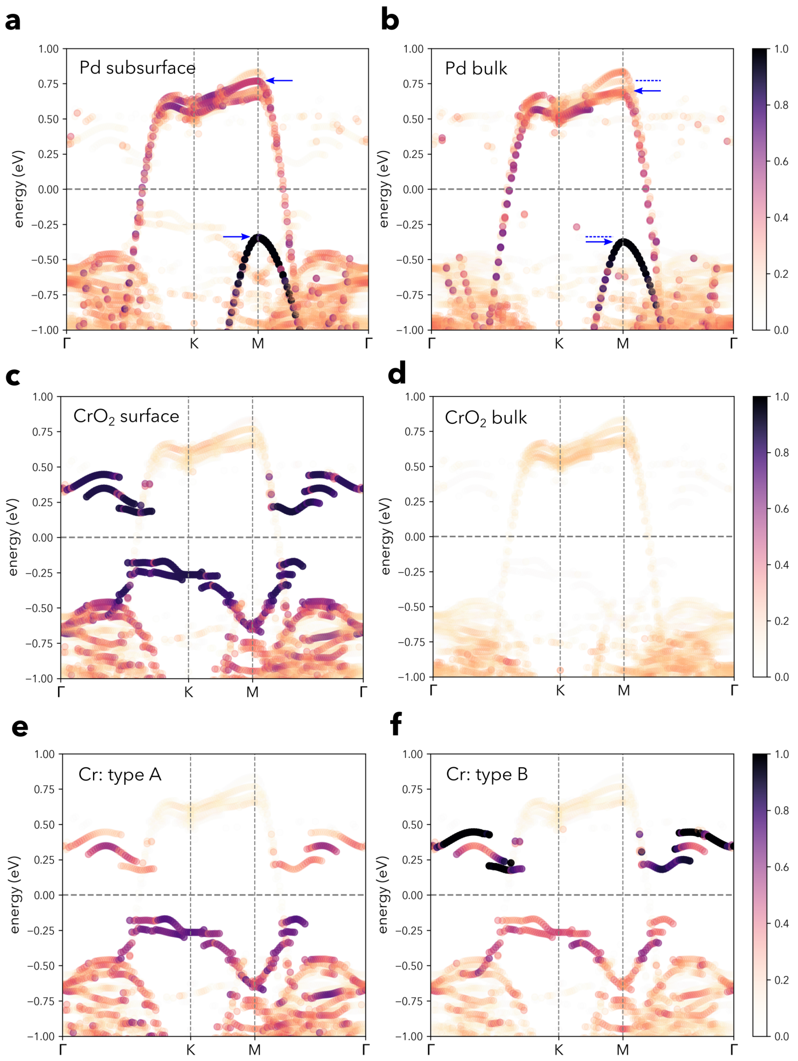

Supplementary Figure S13: **Layer-resolved band structure** (**a**) Band structure of the subsurface Pd layer and (**b**) of the subsequent Pd layer below it, that we refer to as the bulk Pd layer. (**c**) Band structure of the surface and (**d**) the bulk CrO_2 layer. The band structure of the surface CrO_2 layer is further projected onto two types of Cr atoms in (**e**) and (**f**).

any change in the ground-state spin order caused by the scan with $V = 200\text{mV}$ (Fig. S14b), in Fig. S14d we show the difference image formed by subtraction of the cropped region in Fig. S14c by that in Fig. S14a. The difference image shows drastic change in the charge order after the scan with $V = 200\text{mV}$.

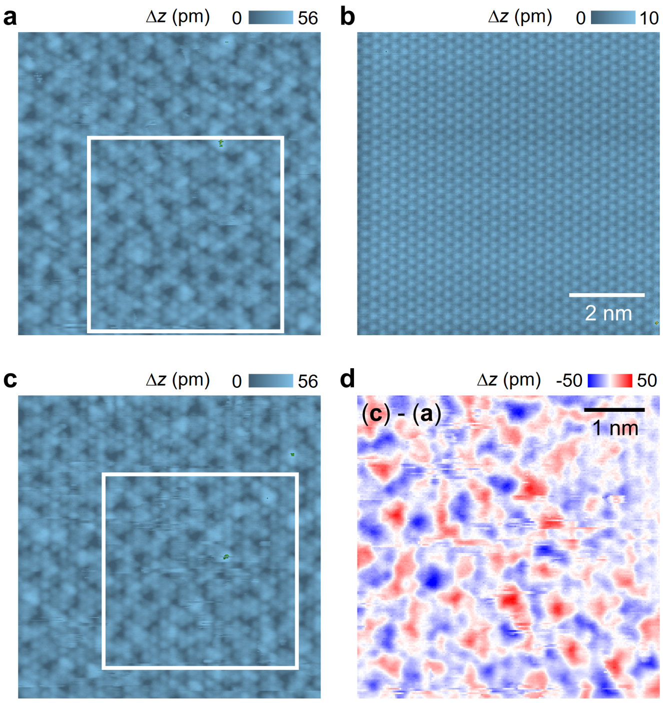

Supplementary Figure S14: **Bias-voltage induced change in the charge order.** **a**, STM topographic image of the CrO_2 terminated surface recorded at low bias voltage $V = 5\text{mV}$. **b**, Image taken at higher voltage $V = 200\text{mV}$ after (**a**). **c**, low voltage image ($V = 5\text{mV}$) taken after (**b**). Image size: $(8\text{nm})^2$. Scam parameters: (**a**,**c**) $V = 5\text{mV}$, $I = 150\text{fA}$; (**b**) $V = 200\text{mV}$, $I = 10\text{pA}$. **d**, Difference image obtained by subtraction of the cropped region (marked by a square) in the image in (**c**) from that in (**a**).

Supplementary note 11. Pulse-induced change in the charge order

To investigate if the charge order evolves back to the original ground state configuration after excitation, we performed two sets of experiments. In the first experiment, we continuously imaged the order using very mild scanning parameters, ($V = 5\text{mV}$, $I = 150\text{fA}$), and amid imaging we occasionally applied voltage pulses ($V = 70\text{mV}$, $\Delta t = 2\text{s}$) to trigger changes in the charge order, and monitored its relaxation. To show our experimental results, in Fig. S15, a to c, we show the image taken before the application of any voltage pulse (Fig. S15a), that taken 312 minutes after the application of the first voltage pulse (Fig. S15b), and that recorded 129 minutes after the application of the second voltage pulse (Fig. S15c). The images look very different from each other. To visualize the changes, we also show their difference images in Fig. S15, d to f, showing drastic changes in the order caused by the voltage pulse(s).

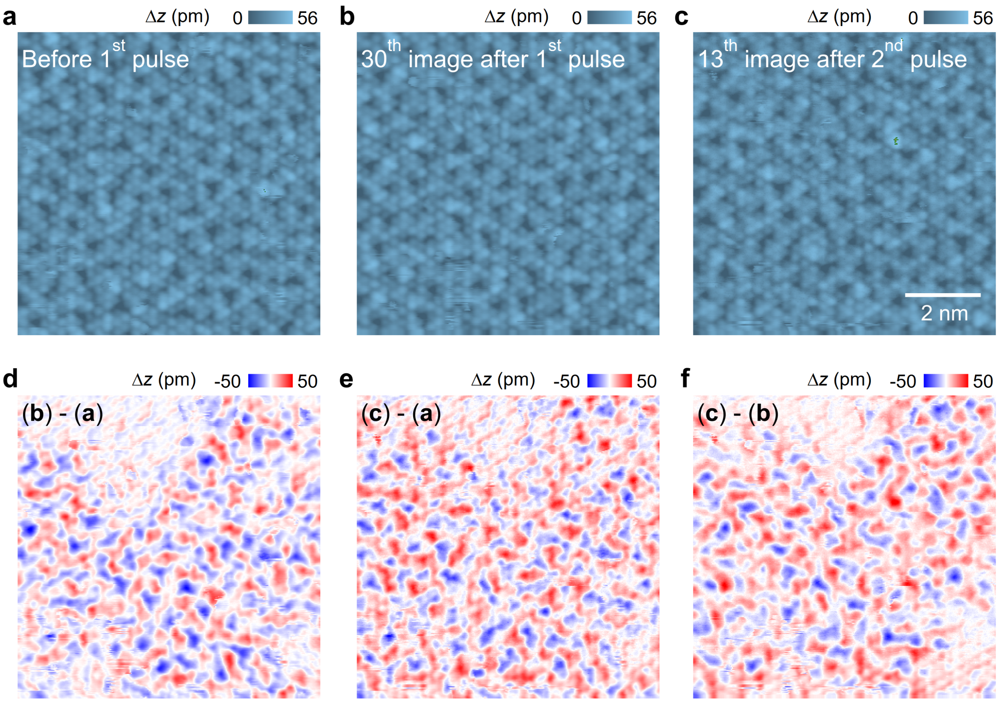

Supplementary Figure S15: **Pulse induced change in the short-range order.** **a**, Atomically resolved STM image of the CrO_2 terminated surface, taken before application of any voltage pulse ($V = 70\mathrm{mV}$, $\Delta t = 2\mathrm{s}$) to the centre of the imaged region. **b**, The 30th image recorded 312 minutes after application of the first voltage pulse. **c**, The 13th image recorded 129 minutes after application of the second voltage pulse. Image size: $(8\mathrm{nm})^2$. Scan parameters: $V = 5\mathrm{mV}$, $I = 150\mathrm{fA}$. All images were recorded at the exact same surface location, under extremely stable, drift-free tunneling condition. **d-f**, Difference images obtained by subtraction between different image pairs formed between (a), (b) and (c) respectively.

References

1. **Chi Ming Yim, Dibyashree Chakraborti, Luke C. Rhodes, Seunghyun Khim, Andrew P. Mackenzie, and Peter Wahl. Quasiparticle interference and quantum confinement in a correlated Rashba spin-split 2D electron liquid. Sci. Adv., 7(15):eabd7361, April 2021.**
2. **Veronika Sunko, H. Rosner, P. Kushwaha, S. Khim, F. Mazzola, L. Bawden, O. J. Clark, J. M. Riley, D. Kasinathan, M. W. Haverkort, T. K. Kim, M. Hoesch, J. Fujii, I. Vobornik, A. P. Mackenzie, and P. D. C. King. Maximal Rashba-like spin splitting via kinetic-energy-coupled inversion-symmetry breaking. Nature, 549(7673):492–496, 2017.**

3. Federico Mazzola, Veronika Sunko, Seunghyun Khim, Helge Rosner, Pallavi Kushwaha, Oliver J. Clark, Lewis Bawden, Igor Marković, Timur K. Kim, Moritz Hoesch, Andrew P. Mackenzie, and Phil D. C. King. Itinerant ferromagnetism of the Pd-terminated polar surface of $PdCoO_2$. PNAS, 115(51):12956–12960, December 2018.
4. F. Mazzola, C. M. Yim, V. Sunko, S. Khim, P. Kushwaha, O. J. Clark, L. Bawden, I. Marković, D. Chakraborti, T. K. Kim, M. Hoesch, A. P. Mackenzie, P. Wahl, and P. D. C. King. Tuneable electron–magnon coupling of ferromagnetic surface states in $PdCoO_2$. npj Quantum Materials, 7(1):20, 2022.
5. Hiroshi Takatsu, Gwilherm Nénert, Hiroaki Kadowaki, Hideki Yoshizawa, Mechthild Enderle, Shingo Yonezawa, Yoshiteru Maeno, Jungeun Kim, Naruki Tsuji, Masaki Takata, Yang Zhao, Mark Green, and Collin Broholm. Magnetic structure of the conductive triangular-lattice antiferromagnet $PdCrO_2$. Phys. Rev. B, 89:104408, Mar 2014.
6. V. Sunko, F. Mazzola, S. Kitamura, S. Khim, P. Kushwaha, O. J. Clark, M. D. Watson, I. Marković, D. Biswas, L. Pourovskii, T. K. Kim, T.-L. Lee, P. K. Thakur, H. Rosner, A. Georges, R. Moessner, T. Oka, A. P. Mackenzie, and P. D. C. King. Probing spin correlations using angle-resolved photoemission in a coupled metallic/Mott insulator system. Sci. Adv., 6(6):eaaz0611, February 2020. Figures reproduced under CC-BY license.
7. Frank Lechermann. Hidden Mott insulator in metallic $PdCrO_2$. Phys. Rev. Materials, 2(8):085004, August 2018.
8. Frank Lechermann. From basic properties to the Mott design of correlated delafossites. npj Comput. Mater., 7(120):1–17, July 2021.
